# Clinicopathologic features and surgical management of primary umbilical melanoma: a case series

**DOI:** 10.1186/s13104-015-1096-x

**Published:** 2015-04-15

**Authors:** Gianluca Di Monta, Corrado Caracò, Ugo Marone, Antonio Maria Grimaldi, Anna Maria Anniciello, Massimiliano Di Marzo, Ester Simeone, Stefano Mori

**Affiliations:** Department of Surgery “Melanoma, Soft Tissues, Head and Neck, Skin Cancers”, National Cancer Institute “G. Pascale”, Via M. Semmola, 80131 Naples, Italy; Unit of Melanoma, Cancer Immunotherapy and Innovative Therapy, National Cancer Institute “G. Pascale”, 80131 Naples, Italy; Department of Pathology, National Cancer Institute “G. Pascale”, 80131 Naples, Italy

**Keywords:** Primary umbilical melanoma, Umbilical tumor, Sentinel lymph node biopsy

## Abstract

**Background:**

Primary umbilical melanoma is an uncommon tumor that is poorly described in the medical literature. The umbilical region is a particular anatomic site owing to the presence of embryonal remnants, which can be a potential metastatic pathway, as well as the braided lymphatic network drainage. Hence, primary malignant neoplasms affecting the umbilicus require a different and more radical surgical approach compared with other melanomas.

**Case presentation:**

In this report, we describe a series of three patients of Caucasian ethnicity who presented with primary umbilical melanoma at the National Cancer Institute of Naples, Italy. All patients underwent wide excision of the tumor including the underlying peritoneum. No surgical complications, either immediate or delayed, were observed in any of the patients. Sentinel lymph node biopsy was negative in two cases. Two of the patients developed metastatic disease and died after systemic medical therapy. The other patient is currently in follow-up, and remains disease-free after 21 months.

**Conclusions:**

The umbilicus has vascular and embryological connections with the underlying peritoneum, so that early visceral involvement is more likely to occur with primary umbilical melanomas. As such, tumor resection including the underlying peritoneum is required to avoid local relapse, whilst sentinel lymph node biopsy appears to be of poor diagnostic value.

## Background

Primary umbilical melanoma is an uncommon disease, with only 23 cases described in the medical literature since it was first reported in 1916 [1] (Table 1). The umbilical region represents an anatomic site with unique characteristics, owing to the presence of embryonal remnants which provide a potential metastatic pathway, as well as the braided lymphatic drainage network. Hence, primary malignant neoplasms that affect the umbilicus require a different surgical approach compared with other melanomas [2]. In this report, we describe three patients who presented with primary umbilical melanoma at the National Cancer Institute of Naples, Naples, Italy, between May 2010 and April 2011.

**Table 1:** **Review of literature**

**Author**	**Year**	**Number of patients**	**Treatment**
Cullen TS [5]	1916	**3**	Excision
Graev M [6]	1957	**1**	Excision
Hughes J [7]	1963	**1**	Excision
Steck WD [8]	1965	**4**	Excision
Breuninger H [9]	1996	**7**	Excision and plastic reconstruction
Colonna MR [10]	1999	**2**	Excision and plastic reconstruction
Meine JG [2]	2003	**1**	Excision and plastic reconstruction
Campos-Muñoz [11]	2007	**1**	Excision, SLNB and plastic reconstruction
Mangas C [12]	2008	**1**	Excision and plastic reconstruction
Cecchi R [3]	2009	**1**	Excision and SLNB
Zaccagna A [1]	2011	**1**	Excision, SLNB and plastic reconstruction
Current study	2014	**3**	Excision, SLNB and plastic reconstruction
**Total**		**26**	

## Case presentation

### Case 1

**Figure 1:**
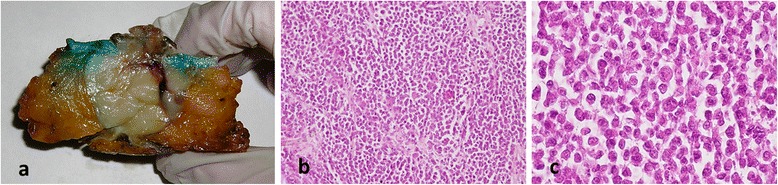
Case 1. **(a)** Specimen cut in half: Umbilicus cavity is completely filled by tumor **(b)** poorly differentiated cells, that look primitive, disorganized and immature (hematoxylin and eosin, ×10) **(c)** diffused anisocytosis, anisonucleosis, altered nuclear-cytoplasmic ratio and evident nucleoli (hematoxylin and eosin, ×60).

**Figure 2:**
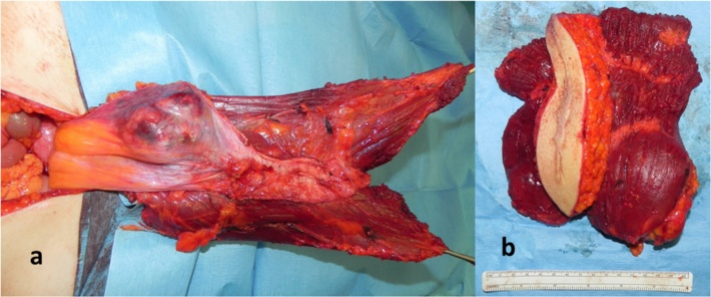
Case 1 local recurrence. **(a)** Local recurrence involving the left rectus muscle up to the peritoneum **(b)** removal of both rectus muscles including the overlying skin bearing the scar of previous excision.

A 33-year-old Caucasian female presented with a coarse nodular pinkish-brown swelling that covered the natural umbilical cavity. Diagnosis of a nodular melanoma was based on clinical findings, with histological investigation showing a Breslow thickness of 40 mm, ulceration, mitotic rate of 6/mm^2^ and no tumor-infiltrating lymphocytes (Figure 1). Whole-body fluorodeoxyglucose positron emission tomography/computed tomography (FDG PET/CT) was negative for distant disease. The patient underwent pre-operative Tc^99^ lymphoscintigraphy according to standard procedure and subsequent removal of the umbilical region, which consisted of the tumor with 2 cm margins including the underlying peritoneum. Sentinel lymph node biopsy (SLNB) in the left inguinal and bilateral axillary regions, as indicated by lymphoscintigraphy, were negative for metastases. Six months after surgery, there was local recurrence involving the posterior aspect of the left rectus muscle up to the peritoneum. The patient underwent surgical removal of both rectus muscles including the local recurrence (Figure 2), with the abdominal wall being reconstructed with non-absorbable Marlex mesh.

However, the patient subsequently developed multiple inoperable nodal and abdominal metastases. A BRAF mutation test was positive and, because BRAF mutation is associated with enhanced and selective sensitivity to MEK inhibition when compared to either wild-type cells or cells with a RAS mutation, the patient was enrolled in an experimental clinical protocol involving treatment with a selective MEK inhibitor (MEK162 15 mg orally). After four months, an ^18^FDG PET/CT scan showed a significant response to treatment with minor intestinal ^18^FDG accumulation in peritoneal thickening close to the bladder (maximum standardized uptake value [SUV_max_] of 3.6), and the disappearance of lymphadenopathy and swellings in the subcutaneous fat pad of the abdominal wall. Therapy was continued for three months, until disease progression was observed on a total body CT scan.

Considering the BRAF mutation-positive status of the patient, treatment was switched to oral vemurafenib 240 mg, a selective BRAF inhibitor. However, this was stopped after 8 weeks due to clinical disease progression. The patient then began a new line of therapy with ipilimumab 3 mg/kg as part of an Italian expanded access programme. Six months later the patient received her fourth administration of ipilimumab, and was completely free of ascites and/or bowel symptoms. The patient died 28 months after initial diagnosis of the disease.

### Case 2

**Figure 3:**
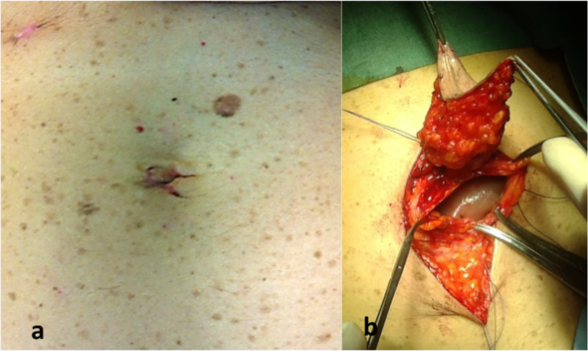
Case 2. **(a)** Pre-operative view **(b)** wide excision comprising the underlying peritoneum.

A 50-year-old Caucasian female had previously undergone removal of a pigmented lesion of the umbilical region at a hospital other than our institute, with histological findings of superficial spreading melanoma, Breslow thickness 2.5 mm, ulceration, mitotic rate of 6/mm^2^ and brisk tumor-infiltrating lymphocytes. After referral to our centre, the umbilical region, including the scar from the initial excision of the lesion, with a 2 cm lateral margin including the underlying peritoneum was surgically removed (Figure 3). The reconstruction of the umbilicus was carried out by means of two local transposition flaps transferred towards the *linea alba*. SLNB in the right inguinal basin was negative for metastases. The patient remains disease-free after 21 months of follow-up.

### Case 3

**Figure 4:**
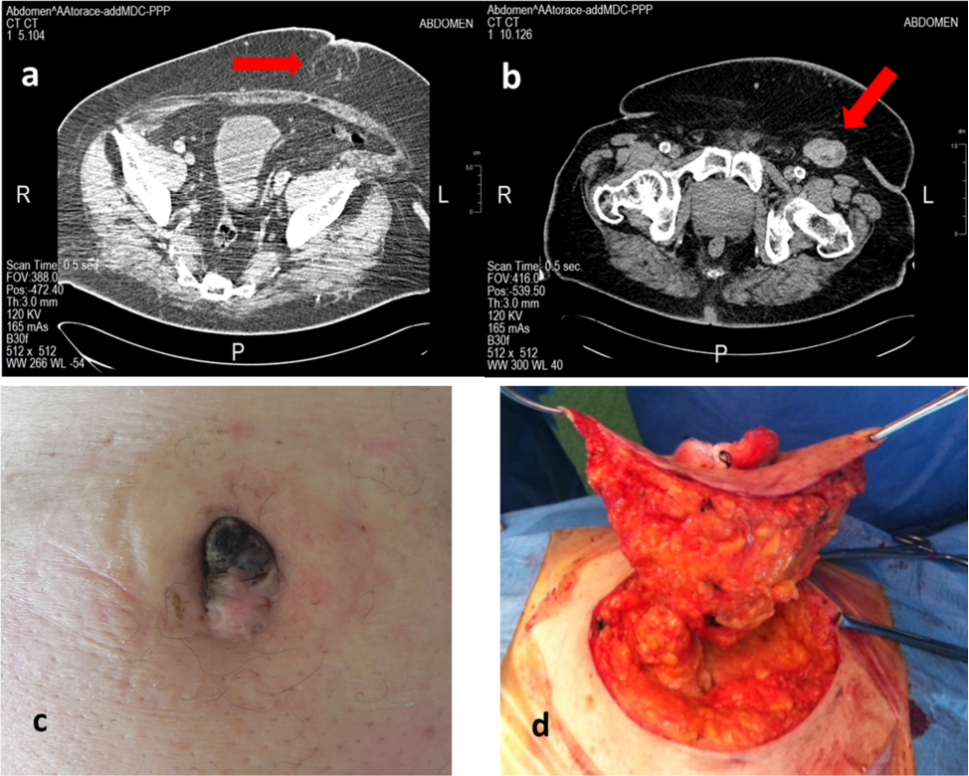
Case 3. **(a)** Computed tomography image showing nodular swelling in the umbilical region **(b)** Computed tomography image of left groin lymphadenopathy of 54 x 28 mm **(c)** pre-operative view **(d)** wide excision comprising the underlying peritoneum.

A 77-year-old Caucasian male had previously undergone removal of a pigmented lesion in the umbilical region at another hospital. Histology showed an ulcerated cutaneous melanoma of 3 mm thickness and mitotic rate of 6/mm^2^. No further treatment was administered until, twelve months later, the patient presented with a swelling underlying his umbilicus. Clinical and instrumental examinations showed a coarse nodular swelling in the umbilical region, most likely local disease recurrence, and major lymphadenopathy of 54 × 28 mm in the left groin, which was positive in pre-operative cytology. The patient underwent left superficial and deep groin dissection and omphalectomy, with 2 cm of lateral margin extended up to the underlying peritoneum (Figure 4). The patient then underwent medical treatment of subsequent bone and liver metastases but died eight months later due to disseminated disease.

## Discussion

Melanoma of the umbilical region is a rare and particularly aggressive neoplastic disease. Primary umbilical malignancies are very poorly described, consisting of melanomas, basal cell and squamous cell carcinomas. The umbilicus has vascular and embryological connections with the underlying peritoneum, with the urachus, vitelline artery and round ligament of the liver representing the main metastatic spread pathway. Primary umbilical melanoma determines a possible early visceral involvement and so should be approached with a wide excision of the entire umbilical structure, including the attachment to the underlying peritoneum [3].

In the three patients reported here, the first died 12 months after the second surgery. The second patient is currently in follow-up, and remains disease-free after 21 months. In the third patient, bone and liver metastases developed a year after the diagnosis of primary umbilical melanoma and led to the patient’s death. No surgical complications, either immediate or delayed, were observed in any of the patients. The medical oncology staff treated two of the three patients for systemic disease.

The difference between primary umbilical melanoma and metastatic tumors of the umbilicus should be noted. Metastatic tumors account for approximately 80-88% of malignancies occurring in the umbilical area [3,4]. The classic example is the “Sister Mary Joseph nodule,” representing a metastasis from the gastrointestinal tract. In a review of 77 umbilical malignancies at a single centre, the most common primary sites with metastasis to the umbilicus were the ovary, endometrium, and pancreatobiliary tree in women, and the genitourinary tract, pancreatobiliary tree, and gastrointestinal tract in men [2-4].

SLNB for primary umbilical melanomas are very rarely reported. The umbilicus has a lymphatic drainage involving axilla and inguinal areas or, more rarely, internal mammary, iliac or intra-abdominal lymph nodes [1]. In our patients, lymphoscintigraphy showed the presence of the sentinel lymph node in both armpits and left groin in the first patient and in the right groin in the second patient. As such, the potential role of SLNB of umbilical tumors represents an unanswered question but at present has to be considered of poor diagnostic value.

## Conclusions

Primary umbilical melanoma should be considered a different pathology from common cutaneous melanoma. Tumor excision *en bloc* with the underlying peritoneum is required to avoid local relapse. Immediate reconstruction of the neo-umbilicus with local flaps should be carried out at the same time, while respecting the aggressive nature of the neoplasm. SLNB appears to be of poor diagnostic value.

## Consent

Written informed consent was obtained from the next of kin of both dead patients and from the patient still alive for publication of this Case Report and accompanying images. A copy of the written consent is available for review by the Editor-in-Chief of this journal.

## Abbreviations

FDG PET/CT: Fluorodeoxyglucose positron emission tomography/computed tomography
SLNB: Sentinel lymph node biopsy
SUV_max_: Maximum standardized uptake value
